# Persistent maternal age effects on male offspring fitness traits in a wild mammal population

**DOI:** 10.1093/evlett/qraf021

**Published:** 2025-07-22

**Authors:** Sanjana Ravindran, Kynan L Delaney, Xavier Bal, Jill G Pilkington, Josephine M Pemberton, Jacob A Moorad, Hannah Froy, Daniel H Nussey

**Affiliations:** Institute of Ecology and Evolution, University of Edinburgh, Edinburgh, EH9 3FL, United Kingdom; Institute of Ecology and Evolution, University of Edinburgh, Edinburgh, EH9 3FL, United Kingdom; Institute of Ecology and Evolution, University of Edinburgh, Edinburgh, EH9 3FL, United Kingdom; Institute of Ecology and Evolution, University of Edinburgh, Edinburgh, EH9 3FL, United Kingdom; Institute of Ecology and Evolution, University of Edinburgh, Edinburgh, EH9 3FL, United Kingdom; Institute of Ecology and Evolution, University of Edinburgh, Edinburgh, EH9 3FL, United Kingdom; Institute of Ecology and Evolution, University of Edinburgh, Edinburgh, EH9 3FL, United Kingdom; Institute of Ecology and Evolution, University of Edinburgh, Edinburgh, EH9 3FL, United Kingdom

**Keywords:** parental age effects, lifetime reproduction, lifespan, Lansing effect, mammals, wild

## Abstract

Effects of parental age on juvenile survival are well documented, but whether parental age has long-term consequences for the fitness of surviving offspring remains poorly understood. This is particularly the case for polygynous mammals, where differential impacts on sons versus daughters are predicted. Here, we investigate the effects of maternal and paternal age on offspring first-year survival, longevity, lifetime reproduction, and annual reproduction in a wild Soay sheep population. We find that younger and older mothers produced offspring that were less likely to survive their first year than middle-aged mothers, and this effect was independent of offspring sex. However, among offspring that survived their first year, adult lifespan and lifetime reproductive success were only influenced by maternal age in sons and not in daughters. Increased adult reproductive success in sons of middle-aged mothers, compared to young and old mothers, was not driven by maternal age effects on offspring reproductive ageing patterns, but potentially by consistent effects on offspring average annual reproductive performance. There was weak evidence of a paternal age effect on offspring longevity but no effect on other offspring traits. Our study shows long-lasting, sex-dependent maternal age effects on offspring fitness traits in the wild, adding to the growing body of literature that highlights the potential importance of intergenerational effects in natural populations.

## Introduction

The age of parents at conception can have a profound impact on the phenotypic traits of their offspring (Monaghan et al., 2020; Gillespie et al., 2013). One explanation for this is the important role that parents play in shaping the environment that offspring experience during development (Monaghan, 2008; Uller, 2012). Evidence of parental age effects on offspring life-history traits has been previously documented in plants, invertebrates, and vertebrates (Ashby & Wangermann, 1949; Bell, 1918; Berkeley et al., 2004; Gavrilov & Gavrilova., 1997; Kroeger et al., 2020; Lippens et al., 2017; Priest et al., 2002; Schroeder et al., 2015; Fay et al., 2016).

Numerous studies in wild vertebrates have shown that maternal age influences offspring development with reduced birth weights and juvenile survival rates reported in offspring born to young and old mothers (Clutton-Brock et al., 1987; Côté & Festa-Bianchet, 2001; Daunt et al., 2001; Descamps et al., 2008; Ericsson et al., 2001; Hammers et al., 2021; Hayward et al., 2013; Hoffman et al., 2010; Keech et al., 2000; Nussey et al., 2009). Such widely reported quadratic maternal age effects can be explained by inexperienced young mothers and senescent individuals in poor condition being unable to provide adequate resources and care to their offspring, compared to adults of intermediate age (Beamonte-Barrientos et al., 2010; Curio, 1983; Lemaître & Gaillard, 2017). Evolutionary genetic theory suggests that rapid declines in age-specific selection for maternal effects could explain negative maternal age effects on early-life offspring traits, especially at older maternal ages (Moorad & Nussey, 2016). However, formal theory that explains the evolution of parental age effects on later-life offspring traits is currently lacking.

There is increasing evidence that parental age shapes the long-term fitness of offspring. The so-called “Lansing effect”—the observation of reduced longevity in offspring of older mothers—has mounting, albeit still mixed, support (Bell, 1918; Lansing, 1947; Priest et al., 2002; Tarín et al., 2005; Plaistow et al., 2015; reviewed in Monaghan et al., 2020; meta-analysis by Ivimey-Cook et al., 2023). Furthermore, studies in humans, wild mammal, and bird populations have found that offspring born to older mothers have lower lifetime reproductive performance (Bouwhuis et al., 2015; Gillespie et al., 2013; Kroeger et al., 2020; Reichert et al., 2020; Rödel et al., 2009; Schroeder et al., 2015). Whether maternal age effects on offspring fitness act via survival, reproduction, or both, and whether they influence offspring mean adult fitness or alter offspring demographic ageing patterns has rarely been studied. Here, we use multigenerational data collected over 35 years from a wild mammal population to test how maternal age influences these different aspects of offspring fitness.

Studies in birds, insects, and mammals reported that older fathers produce lower quality offspring in both lab and natural environments (García-Palomares et al., 2009; Noguera et al., 2018; Fay et al., 2016; Torres et al., 2011; Vuarin et al., 2021; Wylde et al., 2019; but see Cholewa et al., 2021; Cooper et al., 2020). Even in species without paternal postnatal care, recent studies have demonstrated the role of epigenetic and environment-dependent paternal effects on offspring performance (Bonduriansky & Head, 2007; Crean et al., 2013; Vega-Trejo et al., 2018; Zajitschek et al., 2014). Furthermore, in polygynous species where males mate frequently with multiple females within a single mating season, sperm production may be relatively constant and can result in an accumulation of deleterious mutations with age (Pizzari et al., 2008; Ruiz-López et al., 2010). This could translate to potential fitness consequences for offspring sired by older fathers in such systems. Here, we investigate whether paternal age influences offspring fitness in a polygynous ungulate species that lacks paternal postnatal care.

Across both experimental and field studies, sex-specific patterns of parental effects on offspring traits have been observed (Bouwhuis et al., 2015; Schroeder et al., 2015; Sparks et al., 2022; Hellmann et al., 2020; Qvarnstrom & Price., 2001; Braithwaite et al., 2017; Metzger & Schulte., 2016). For example, in wild house sparrows and common terns, parental age effects manifested only in offspring of the same sex as the parent (i.e., maternal effects on female offspring, paternal effects on male offspring; Bouwhuis et al., 2015; Schroeder et al., 2015). However, in Seychelles warblers, maternal age influenced both male and female offspring traits (Sparks et al., 2022). In sexually dimorphic species where males attain larger body size, sons are expected to entail higher physiological costs for mothers due to their greater nutritional needs compared to females (Clutton-Brock, 1991). The increased resource requirement of males coupled with faster growth rates and investment in secondary sexual traits could make them more vulnerable to poor conditions experienced early in life than females. Thus, in polygynous species where the distribution of reproductive success in males is more variable and skewed compared to females, we would predict male offspring fitness to be more sensitive to effects of parental age compared to daughters (Hewison & Gaillard, 1999; Trivers & Willard, 1973). Previous studies in wild or semi-captive polygynous populations have focused on evaluating maternal age effects on female offspring traits (Kroeger et al., 2020; Rödel et al., 2009; Reichert et al., 2020). The prediction that both maternal and paternal age effects should be stronger on male compared to female offspring lifetime fitness traits remains to be tested in polygynous mammals in the wild.

Here, we investigate how parental age influences lifespan and lifetime fitness of sons and daughters in a sexually dimorphic species, the Soay sheep (*Ovis aries*). Previous studies in this wild population revealed maternal age effects on offspring birth weight, first-year survival, and neonatal antibody levels (Hayward et al., 2013; Sparks et al., 2020). We predict similar quadratic patterns of maternal age effects on offspring fitness traits and hypothesize offspring sex-dependent associations, predicting sons to be impacted more than daughters. Since mothers provide postnatal care but not fathers, we predict any effect of father’s age, if present, to be weaker in magnitude than (but in the same direction as) the maternal age effect in this system.

## Methods

### Study system

Individual-based monitoring of the Soay sheep resident in the Village Bay area of Hirta, St. Kilda, has been ongoing since 1985. The mating season begins in late October–early November with males competing to impregnate estrous females. Males consort with females for several hours forming consort pairs and mating repeatedly. Males can mate in their first autumn but are poor competitors in the rut and less likely to sire offspring compared to adult males (Clutton-Brock & Pemberton, 2004). Females can be sexually mature and come into estrus during their first rut (aged 6–7 months), but lambs born to yearling ewes are less likely to survive their first year. From next year onward, almost all adult females come into estrus and most conceive, although there is evidence of reproductive senescence past 7 years of age (Hayward et al., 2013). Lambs are born during late March–April after a ∼5-month gestation period. Although most lambs are singletons, around 2%–23% of births are twins, depending on the year (Clutton-Brock et al., 1991). Around 95% of the lambs born in the study area are caught and uniquely tagged for identification making them of known age (Clutton-Brock & Pemberton, 2004; Robertson et al., 1992). Over 85% of sheep mortality occurs during February–April. Regular censusing along with mortality searching and high resighting probabilities (0.93 for females and 0.82 for males; Catchpole et al., 2000) means survival and reproduction of tagged individuals across their lifetimes are known (Clutton-Brock & Pemberton, 2004). A multigenerational genetic pedigree has been inferred using a subset of 431 single nucleotide polymorphisms with high minor allele frequency and low linkage disequilibrium, enabling the accurate assignment of both parents to offspring (Bérénos et al., 2014; Huisman, 2017).

### Dataset and statistical analyses

For offspring first-year survival, our dataset was comprised of 4,807 individuals born between years 1986 and 2019. Our dataset for offspring lifetime traits and annual breeding performance was composed of 1,286 offspring individuals born between years 1986 and 2015. All offspring were monitored between years 1986 and 2020. In this dataset, there was no evidence of age-assortative mating, and since the correlation between maternal and paternal ages was weak (Pearson’s correlation coefficient, *r* = 0.051, 95% CI: −0.004 to 0.105), we were able to separate the effects of maternal and paternal age and include the ages of both parents as continuous predictors in all our statistical models (Froy et al., 2017). This allowed us to examine the independent effects of maternal and paternal age on offspring traits and excluded the possibility of parental age effects acting via influences of the partner’s age.

### Offspring fitness traits

We first evaluated the effect of parental age on offspring first-year survival (to May 1 of the year following birth, *n* = 4,807). We then investigated parental age effects on adult traits, including only offspring that survived beyond one year of age (*n* = 1,286). We considered three offspring lifetime traits: longevity (in years) beyond the first year (i.e., adult lifespan—determined based on the recorded year of death or when individuals were last seen in a census); lifetime breeding success (LBS, number of offspring born to or sired by an individual across their lifespan); and lifetime recruitment success (LRS, number of offspring that survived to May 1 of the year following their birth across an individual’s lifespan). Finally, we modeled annual breeding performance separately for male and female offspring: male annual breeding success (number of offspring sired); female annual breeding probability (0 = did not breed, 1 = bred); and female annual twinning probability (conditional on having bred, singleton = 0, twin = 1). Further details regarding sample sizes and a detailed description of offspring traits are available in the Supplementary Materials (Supplementary methods; Tables S1 and S2; Figures S1 and S2).

### Modeling parental age effects

We evaluated whether parental age effects were present in all seven offspring traits using generalized additive mixed models (GAMMs) implemented in the R package “mgcv” v.1.8.33 (Wood, 2012). We fitted a GAMM model for each offspring trait (response variable), applying a restricted maximum likelihood (REML) approach for smoothness estimation. Fitting GAM models allowed us to make no assumptions about the shape of the age effect.

For all offspring traits, we initially fitted smooth terms for both maternal and paternal age but only found support for nonlinearity (effective degrees of freedom, EDF > 1) in the maternal age effect. We therefore fitted a linear parametric term to model paternal age. For the maternal age smooth terms, we constrained the spline basis dimension, *k*, to a relatively small value (*k* = 4) to avoid overfitting. This meant that three basis functions were used to model nonlinearity in maternal age effects (Wood, 2017). Since individuals have much lower chances of successfully producing and recruiting offspring in their first year, maternal and paternal age classes distinguishing offspring born to yearling vs. adult parents (a two-level factor) were also included as fixed effects. Offspring first-year survival, female annual breeding probability, and annual twinning probability were fitted as a binomial response with logit link function. Offspring longevity, LBS, LRS, and male offspring annual breeding success were fitted with negative binomial errors and a log link function.

Since litter size at birth may influence the amount of maternal investment and overall survival and fitness of offspring, we included a factor accounting for whether an offspring was a twin or a singleton as a fixed effect. We also included offspring sex (two-level factor for males and females) as a fixed effect in our models to account for sex differences in the response variable. Maternal and paternal longevity were fitted as continuous covariates in the models to account for selective disappearance effects of parents. By doing so, we could interpret the parental age terms as within-individual effects (Nussey et al., 2008; Van de Pol & Verhulst., 2006). Maternal and paternal identity along with offspring birth year were included as random effects in all models to account for non-independence of offspring born to the same parents and temporal environmental variation across birth cohorts. Furthermore, in all annual models, we also included the age of the offspring as a fixed effect ,fitted as a smooth term (*k* = 4). These annual models also included the identity of the offspring and year of observation as random effects to account for repeated measures of the offspring individual across multiple years.

We tested for offspring sex-specific variation in parental age effects on offspring first-year survival, adult lifespan, LBS, and LRS by comparing the base model (no interaction terms) with models that contained an interaction between offspring sex and either maternal age, paternal age, or both. For this purpose, GAMMs were refitted using ML smoothness estimation to obtain comparable Akaike Information Criteria (AIC) scores (Zuur et al., 2009). As an additional assessment of model performance, we also report the change in the residual deviance and degrees of freedom for each model compared to the base model. If there was support for an interaction between parental age and offspring sex (i.e., ∆AIC > 2; Anderson & Burnham, 2002), we explicitly tested whether the parental age spline for male offspring was significantly different from the spline for female offspring by running another GAMM recoding offspring sex as an ordered factor, which allowed us to obtain the difference in smooths between male and female offspring (Simpson, 2017). Models were refitted using REML when reporting model estimates.

Since GAMMs model associations between the response and predictor variables as a function of many nonlinear smooth terms, any significant parental effects may only influence offspring traits over a specific range of parental age values. To identify the regions where parental age effects are significant, we used a finite difference method implemented in the “*gratia*” R package to estimate the first derivatives (or slopes) of the fitted splines and their associated 95% simultaneous confidence intervals at regular intervals along the function (Simpson & Singman, 2018). The first derivative (i.e., rate of change at each point evaluated) can be considered statistically significant if the 95% confidence intervals do not overlap zero.

Details of models investigating whether parental age effects on offspring lifetime traits are independent of offspring longevity, parental partner age, and whether parental age influences the reproductive ageing pattern of offspring are provided in the Supplementary Materials.

## Results

### Offspring first-year survival

There was a nonlinear effect of maternal age on offspring first-year survival (EDF = 2.923; *χ^2^* = 110.35; *p* < .001; Figure S3A; Table S4). Offspring first-year survival increased with maternal age up to age 5, after which there was a decline in the survival probability of offspring born to mothers aged 6–10 years (Figure S3B). In contrast, there was no support for any paternal age effect on lamb first-year survival (linear estimate: −0.037; 95% CI: −0.093 to 0.019; Table S4). There was no support for sex differences in parental age effects on offspring first-year survival: including interactions between offspring sex and parental age terms did not improve the explanatory power of the model (∆AIC = +5.590; Table S3). Similarly, we found no evidence for an interaction between maternal and paternal age (∆AIC = +0.669; Table S7, S11).

### Offspring adult lifespan

**Figure 1.**
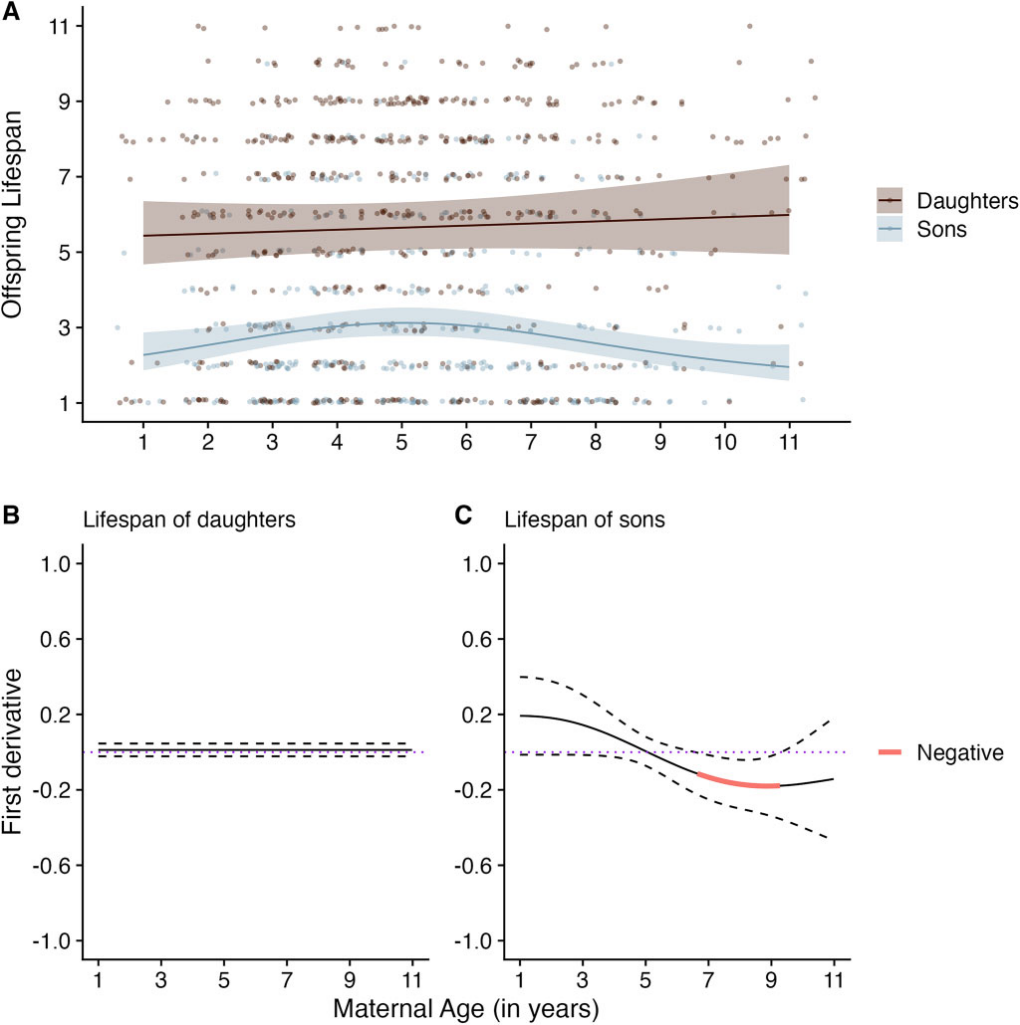
Maternal age effects on longevity of offspring that survived beyond 1 year of age. (A) represents offspring sex-specific effects of maternal age on offspring lifespan. (Model predictions and 95% confidence intervals shown. Jittered raw data only shown for offspring lifespan ranging from 1 to 11 years. Observations with maternal ages >11 were binned into age 11.) (B) and (C) are the simultaneous 95% CIs for the smooth maternal age terms for each offspring sex. Highlighted (red) areas indicate maternal ages where offspring lifespan significantly declines with maternal age.

The best-fitting model indicated that maternal age effects on offspring adult lifespan differed between male and female offspring: The inclusion of a maternal-age-by-offspring sex interaction improved the explanatory power of the base model (∆AIC = −7.617, Table S3; difference in sons compared to daughters: EDF = 2.382; *χ^2^* = 12.325; *p* = .012; Table 1). We found only adult lifespan of sons, not daughters, was associated with maternal age (Figure 1A–C). The finite difference method revealed a statistically significant decline in adult lifespan of sons whose mothers were aged between 7 and 9 years (Figure 1C). There was weak evidence for a negative paternal age effect on offspring adult lifespan, though the 95% CIs spanned zero (linear estimate: −0.037; 95% CI: −0.076 to 0.002; Table 1). There was no evidence of any offspring sex-specific effects of paternal age on offspring lifespan (∆AIC = +1.126; Table S3). In addition, there was no support for an interaction between maternal age and paternal age on offspring adult lifespan (∆AIC = +1.011; Tables S7 and S11).

**Table 1.** Results from the best-fitting generalized additive mixed model (GAMM) estimating parental age effects on offspring lifespan.

Fixed effect	Model		
	Estimate	95% CI	*p*-value
**Intercept**	**1.075**	**0.725, 1.425**	**<.001**
Paternal age	−0.037	−0.076, 0.002	.061
**Offspring sex (male)**	**−0.948**	**−1.067, −0.828**	**<.001**
Twin (1)	−0.151	−0.324, 0.021	.086
**Maternal lifespan**	**0.039**	**0.013, 0.066**	**.003**
**Paternal lifespan**	**0.042**	**0.008, 0.076**	**.017**
Maternal age class (yearling mum)	−0.331	−0.777, 0.116	.147
Paternal age class (yearling dad)	−0.026	−0.304, 0.251	.852
Smooth terms	EDF	*χ* ^2^	*p*-value
s (maternal age)	1.336	0.453	.598
**s (maternal age): offspring sex (male)**	**2.382**	**12.325**	**.012**
s (maternal ID)	0.010	0.008	.994
s (paternal ID)	0.005	0.004	.963
**s (birth year)**	**18.080**	**70.885**	**<.001**

*Note*. This was fitted using negative binomial error distribution. Fixed effects, smoothing splines, and random effects estimates, 95% confidence intervals, and *p*-values are shown. Results presented are on a log link scale (*n* = 1,286 offspring individuals). Statistically significant effects (*p* < .05) are highlighted in bold.

### Offspring LBS and LRS

**Figure 2.**
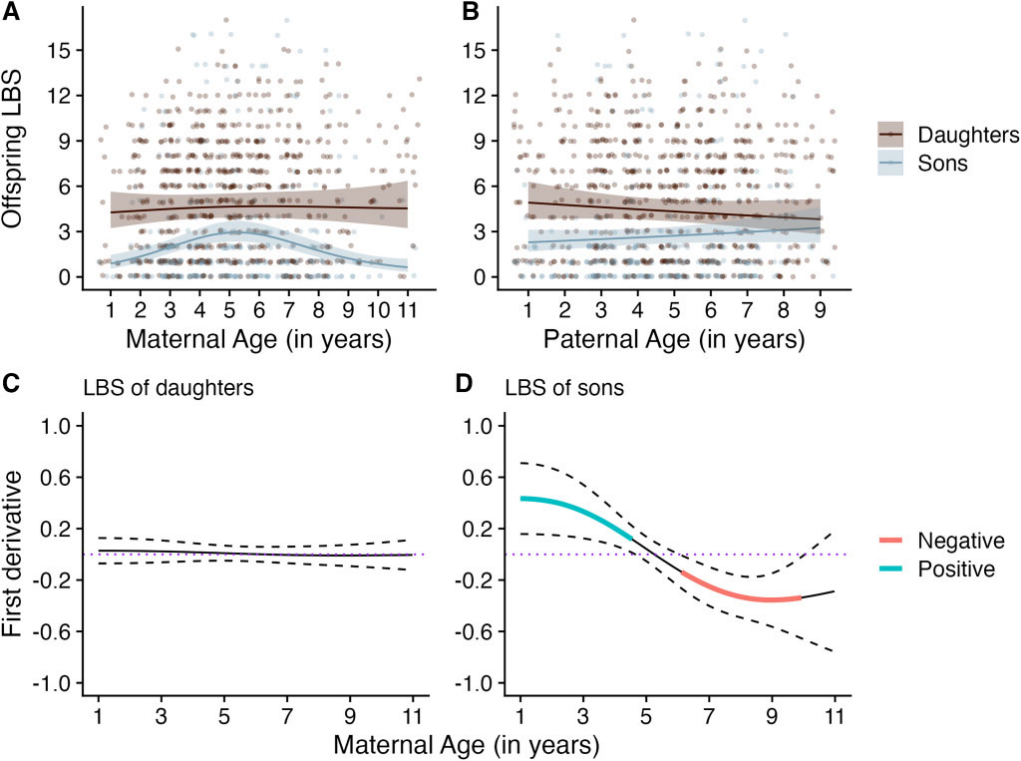
Maternal and paternal age effects on the lifetime breeding success (LBS) of offspring that survived beyond 1 year of age. (A) and (B) represent maternal and paternal age effects in sons and daughters (Table 2). Model predictions and 95% CIs displayed. Jittered raw data only shown for offspring LBS ranging from 0 to 15. Observations with maternal ages >11 were binned into age 11, and paternal ages >9 were binned into age 9. (C) and (D) represent the simultaneous 95% CIs for the smooth maternal age terms for each offspring sex. Highlighted (red or blue) areas indicate maternal ages where offspring LBS significantly increases or declines with maternal age.

We found support for offspring sex-dependent parental age effects on offspring LBS and LRS. The best-fitting model included interactions between offspring sex and both maternal and paternal ages (∆AIC = −11.238 and −11.299 for offspring LBS and LRS, respectively; Table S3). We found sex-specific effects of maternal age on offspring LBS (difference in sons compared to daughters: EDF = 2.596; *χ^2^* = 20.991; *p* < .001, Figure 2A; Table 2) and offspring LRS (EDF = 2.489; *χ^2^* = 12.873; *p* = .011; Figure S4; Table S5). A maternal age effect was observed in male but not female offspring: LBS of sons increased until mothers were 5, then declined between 6 and 10 years of age, but there was no change in daughters’ LBS with maternal age (Figure 2C, D). Similarly, LRS for sons increased with increasing maternal age until prime adulthood (4 years old) and declined between 7 and 9 years (Table S5; Figure S4A, D). This sex-specific effect of maternal age on offspring LBS remained significant after accounting for offspring lifespan (EDF = 2.522; *χ^2^* = 18.690; *p* < .001; Table 2), but this was not the case for offspring LRS (EDF = 1.931, *χ^2^* = 4.428, *p* = .164; Table S5).

**Table 2.** Results from generalized additive mixed model (GAMM) estimating parental age effects on offspring lifetime breeding success.

Fixed effect	Model A			Model B		
	Estimate	95% CI	*p*-value	Estimate	95% CI	*p*-value
**Intercept**	**0.812**	**0.332, 1.292**	**.001**	**−0.636**	**−0.978, −0.294**	**<.001**
Paternal age	−0.030	−0.086, 0.025	.281	0.024	−0.013, 0.062	.204
**Offspring sex (male)**	**−1.024**	**−1.407, −0.641**	**<.001**	**0.319**	**0.036, 0.602**	**.027**
**Twin (1)**	**−0.345**	**−0.570, −0.121**	**.003**	**−0.172**	**−0.333, −0.012**	**.035**
**Maternal lifespan**	**0.064**	**0.028, 0.100**	**.001**	0.022	−0.003, 0.048	.085
Paternal lifespan	0.042	−0.003, 0.086	.069	−0.005	−0.035, 0.025	.740
**Maternal age class (yearling mum)**	**−0.643**	**−1.256, −0.030**	**.040**	−0.427	−0.863, 0.009	.055
Paternal age class (yearling dad)	0.087	−0.266, 0.439	.631	0.075	−0.170, 0.320	.547
**Paternal age:offspring sex (male)**	**0.074**	**0.001, 0.147**	**.046**	0.012	−0.041, 0.064	.664
**Offspring Lifespan**				**0.330**	**0.312, 0.347**	**<.001**
Smooth terms	EDF	*χ* ^2^	*p*-value	EDF	*χ* ^2^	*p*-value
s (maternal age)	1.995	2.277	.517	1.001	0.689	.407
**s (maternal age):offspring sex (male)**	**2.596**	**20.991**	**<.001**	**2.522**	**18.690**	**<.001**
**s (maternal ID)**	**141.146**	**202.579**	**<.001**	**120.831**	**175.221**	**<.001**
s (paternal ID)	36.531	44.791	.095	3.190	3.229	.460
**s (birth year)**	**15.126**	**59.092**	**<.001**	**11.852**	**26.391**	**.005**

*Note*. This was fitted using negative binomial error distribution. Fixed effects, smoothing splines, and random effects estimates, 95% confidence intervals, and *p*-values are shown. Model A is the best-fitting model, and model B includes offspring lifespan as an additional fixed effect. Results presented are on a log link scale (*n* = 1,286 offspring individuals). Statistically significant effects (*p* < .05) are highlighted in bold.

Although there appeared to be differences in the paternal age effect between sons and daughters, the slopes for male and female offspring did not differ from zero since we found the 95% confidence intervals to overlap 0 for both offspring LBS and LRS (slope estimate for sons LBS: 0.044; 95% CI: −0.024 to 0.112; slope estimate for daughters LBS: −0.031; 95% CI: −0.086 to 0.025; Table 2; Figure 2B; Table S6; slope estimate for sons LRS: 0.071; 95% CI: −0.014 to 0.157; slope estimate for daughters LRS: −0.047; 95% CI: −0.116 to 0.023; Figure S4B; Table S5, S6). These offspring sex-specific differences of paternal age on offspring LBS and LRS were also no longer significantly different from each other after accounting for offspring longevity in our models (offspring LBS: 0.012; 95% CI: −0.041 to 0.064 (Table 2); offspring LRS: 0.035; 95% CI: −0.042 to 0.112 (Table S5)). The model selection approach revealed no support for an interaction between maternal and paternal age on either male or female offspring LBS (∆AIC = +3.729 and + 2.020, respectively; Tables S7 and S11) and LRS (∆AIC = +5.566 and + 1.397, respectively; Tables S7 and S11).

### Offspring annual breeding performance

There was weak evidence of a bell-shaped maternal age effect on male offspring annual breeding success (smooth estimate: EDF = 2.189; *χ^2^* = 5.102; *p* = .086; Table S8; Figure S5) but no evidence of a paternal age effect (0.047; 95% CI: −0.030 to 0.123). There was no maternal age effect on the ageing pattern of this offspring trait (i.e., no interaction between maternal age and offspring age; ∆AIC = −1.595; EDF = 1.373; *χ^2^* = 2.707; *p* = .140; Table S8). This suggests weak support for mothers in their prime giving birth to sons who have higher annual breeding success (on average) across their lifespan compared to sons born to younger or older mothers, and no evidence of a maternal age effect on the overall reproductive ageing pattern of offspring.

We found no support for any parental age effects on female offspring annual breeding probability (Model A, Table S9) and weak support for a linear maternal age effect on female offspring twinning probability (smooth estimate: EDF = 1.000; *χ^2^* = 3.131; *p* = .077; Table S10). We also found no support for an interaction between maternal age and offspring age in both these traits (Tables S9 and S10—Model B; ∆AIC for breeding probability model = +1.772 and ∆AIC for twinning probability model = −1.744). This suggests no maternal age effect on the ageing pattern of daughters’ annual breeding or twinning success.

## Discussion

Our results confirm previous evidence of maternal age effects on offspring first-year survival in our study system (Hayward et al., 2013), and reveal longer-term, sex-specific effects on offspring longevity, LBS, and LRS. Offspring born to younger and older mothers had reduced first-year survival, relative to middle-aged mothers, regardless of their sex. Among offspring that survived their first year, young and old mothers produced sons that had reduced lifespan, LBS, and LRS compared to middle-aged mothers, but daughters were not affected by maternal age. Maternal age effects on the LBS of sons were independent of sons’ lifespan. Reduced LBS in sons of young and elderly mothers was likely to be driven by reduced average annual breeding success across the sons’ lifetimes and not through changes in their pattern of reproductive ageing. We found little evidence to support the existence of paternal age effects on offspring traits in this population. Our findings provide strong support for the prediction that maternal age effects should be male-biased in polygynous species, and that paternal age effects should be limited in mammals lacking paternal postnatal care. While many studies in wild vertebrates document parental age effects on early-life offspring traits, here we report long-term impacts of maternal age on both lifespan and lifetime reproductive performance of male offspring.

The Lansing effect, i.e., reduced offspring longevity with increasing maternal age, has been reported across diverse taxa (Bock et al., 2019; Ducatez et al., 2012; Gillespie et al., 2013; Lansing, 1947; Marasco et al., 2025; Preist et al., 2002; Plaistow et al., 2015; Reichert et al., 2020; Tarin et al., 2005; Ivimey-Cook et al., 2023). In wild populations, only three studies, all conducted in bird species, have examined the effects of parental age on both male and female offspring lifespan (Bouwhuis et al., 2015; Schroeder et al., 2015; Sparks et al., 2022). Two of these studies reported a sex dependence: in common terns, older fathers had shorter-lived sons, and in Seychelles warblers, older mothers had shorter-lived daughters (Bouwhuis et al., 2015; Sparks et al., 2022). Here, we found a similar negative association between late maternal age and male offspring lifespan. In our population, male lambs require greater maternal investment and are more sensitive to variation in their natal environment compared to females (Clutton-Brock & Pemberton, 2004). We would expect any effects of maternal condition on offspring quality to be stronger in males and potentially greater in magnitude after 1 year of age when the growth trajectories of male and female lambs diverge markedly (Clutton-Brock & Pemberton, 2004; Clutton-Brock et al., 1985, 1991; Drake et al., 2025). Such a maternal age effect could carry over across the lifetime of individuals resulting in sex-dependent associations with offspring lifespan and overall fitness outcomes (Metcalfe & Monaghan, 2001; Stearns, 1992). In addition to finding a within-individual maternal age effect, we also observed longer-lived parents to have longer-lived offspring, which suggests consistent differences among parent individuals (such as in their genetics or early-life environmental conditions) could also contribute to variation in offspring longevity. Although human studies suggest no robust negative effect of advanced maternal age on the physiological and functional traits of offspring, investigation of maternal age effects on offspring frailty in natural animal populations could improve our understanding of the underlying drivers of the Lansing effect in the wild (Hubbard et al., 2009; Myrskylä & Fenelon, 2012).

Previous studies in humans and wild vertebrate populations have found a negative association between maternal age and offspring lifetime reproduction (Bouwhuis et al., 2015; Gillespie et al., 2013; Schroeder et al., 2015). Other relationships, such as either a concave down (Rodel et al., 2009), concave up (Reichert et al., 2020), a positive (Kroeger et al., 2020), or no association (Bouwhuis et al., 2010), have also been reported. Studies in wild or semi-captive polygynous populations focused specifically on female offspring traits finding maternal age effects on offspring lifetime reproduction (Kroeger et al., 2020; Reichert et al., 2020; Rödel et al., 2009). In contrast to these studies, we found no effect of maternal age on daughters’ LBS in the Soay sheep. Previous studies in wild populations of house wrens and a polygynous bird, the spotless starling, reported maternal investment and maternal condition impacting the fitness of male offspring but not female offspring (Bowers et al., 2015; Rubalcaba & Polo, 2018). In polygynous, sexually dimorphic species such as the Soay sheep, males experience strong intrasexual competition to mate compared to females (Hewison & Gaillard, 1999; Preston et al., 2001, 2005). Reproductive success of males in such populations is determined mainly by their large body size and weaponry traits (McPherson & Chenoweth, 2012; Preston et al., 2012; Rico-Guevara & Hurme, 2019). The ability of male offspring to achieve high postnatal growth rates, attain increased body weight, and invest in secondary sexual traits is likely to be affected by the quality of care and amount of initial resources allocated by their mothers (Festa-Bianchet et al., 2000; Kruuk et al., 1999; Lindström, 1999; Monteith et al., 2018; Preston et al., 2005). Effects of variation in developmental conditions (e.g., maternal condition and provisioning rates) could have a greater impact on the reproductive outcomes of sons more than daughters in this population (Drake et al., 2025). Furthermore, the relationship between maternal age and offspring LBS was independent of offspring longevity (Table 2—Model B). This suggests that small effects of maternal age on offspring annual reproduction could potentially translate to reduced LBS in sons of younger and older mothers over their lifetime (Table S8). The knock-on consequences of early-life, maternal condition-mediated effects on male offspring LBS could influence the age and sex structure and transient growth rate of such populations in complex ways; more work is needed to understand how such long-term parental age effects shape population dynamics in the wild (Gaillard et al., 2000).

Findings from human studies reported negative associations between paternal age and infant birth weight, mortality rates, and offspring longevity (Gourbin &, Wunsch,1999; Kemkes-Grottenthaler, 2004; Reichman & Teitler, 2006). In wild bird populations with biparental care, paternal age effects on juvenile survival, lifespan, and LRS have been observed (Bouwhuis et al., 2015; Fay et al., 2016; Schroeder et al., 2015). The lack of paternal postnatal care in Soay sheep means our finding of no consistent paternal age effects across offspring traits was expected. In older fathers, reduced quality of DNA within sperm due to increased de novo mutation rates or epigenetic changes has been reported to underlie negative paternal age effects on offspring traits (Johnson et al., 2015; Kong et al., 2012). Inheritance of short telomeres from older fathers could also contribute since short telomere lengths (TLs) are associated with lower survival probabilities in vertebrates (Bauch et al., 2019; Wilbourn et al., 2018). However, a previous study in this Soay sheep population found no effect of either paternal or maternal age on offspring telomere length (Froy et al., 2017). It is clear from our analysis that paternal age effects observed in some species do not translate to this wild mammal population lacking paternal care of any sort.

In conclusion, our study highlights the sex-dependent nature of maternal age effects in a polygynous species and the important long-term consequences of such effects for offspring fitness. Many studies have documented maternal age effects to be prevalent and primarily influencing early-life offspring traits. However, studies characterizing the cascading effects of maternal age on offspring lifespan and lifetime reproduction among the sexes in wild mammalian populations remain scarce. Our research has uncovered long-lasting maternal age effects influencing the lifetime fitness of male offspring in a polygynous, sexually dimorphic ungulate species. Investigation into the mechanisms underlying these effects along with the long-term demographic outcomes of such parental age effects will improve our understanding of the impact of parental age effects on variation in life-history strategies and ageing rates in the wild.

## Supplementary Material

qraf021_Supplemental_File

## Data Availability

Data are available on Zenodo (https://doi.org/10.5281/zenodo.15608495), and the analysis scripts are available on GitHub (https://github.com/sanjanaravindran/ParentalAgeEffects-SoaySheep).
