# Supplementary material for: Persistent maternal age effects on male offspring fitness traits in a wild mammal population
Source: Evol Lett. 2025 Jul 22;9(5):511–21. doi: 10.1093/evlett/qraf021 (PMC12492104; doi:10.1093/evlett/qraf021)
Supplement: qraf021_Supplemental_File [file qraf021_supplemental_file.docx]

**Supplementary Materials**

Methods

Dataset

First-year survival (offspring that survive to May 1 of the year following their birth) was a binary trait taking the values 1 if the offspring survived to 1 year of age and 0 if not. Lifespan was estimated as the number of years an individual was alive after May 1 following their year of birth (range of values: 0-14 in female offspring and 0-10 in male offspring). Lifetime breeding success (LBS) was calculated as the number of live offspring born to or sired by an individual across their lifespan (range of values: 0-20 in female offspring; 0-94 in male offspring). Lifetime recruitment success (LRS) was defined as the number of recruits (offspring that survive to May 1 of the year following their birth) an individual produced across their lifespan (range of values: 0-8 in female offspring; 0-33 in male offspring). Male annual breeding success was the number of live offspring sired by an individual in each year of their life, throughout their lifespan (range: 0-22). Female annual breeding probability (0 = did not breed and 1=bred) and annual twinning probability (0=singleton and 1=twin, conditional on having bred) were binary traits.

For offspring first-year survival, we used a dataset comprised of offspring individuals born between 1986 - 2019 (n = 4807 observations from 2463 female and 2344 male offspring) with known mother and father identities and known birth years of parents to ascribe parental age at birth. For offspring lifespan, LBS, LRS and annual breeding performance traits (male ABS, female annual breeding and twinning probability), our analyses were restricted to only those offspring that survived to ≥ 1 year of age. This dataset was comprised of offspring individuals born between 1986 – 2015 with known mother and father identities and known birth years of parents. In total, there were 1286 offspring individuals (754 female and 532 male recruits) born to 607 mothers and 429 fathers. We restricted the dataset to offspring individuals born in or before 2015 which meant that > 90% of offspring individuals in this dataset are already dead and for whom lifespan, LBS and LRS values are complete. For offspring that are alive (as of year 2020), their age at last observation was assumed as their longevity. In addition, > 97% of parents were dead and we therefore did not expect the dataset to be biased towards offspring of shorter-lived mothers and/or fathers. Further details of sample size and general characteristics of datasets available in Supplementary Tables 1-2 and Figures S1-S2.

Statistical Analyses

In all the offspring lifetime traits models, we included parental age classes (distinguishing between yearling and adult parents) as a two-level categorical fixed effect since only 2.33% offspring were born to a yearling mother and 5.91% offspring were born to a yearling father, out of the 1286 offspring individuals in the dataset. For all models, we calculated the Pearson estimate for the dispersion parameter using the Pearson residuals for all models and found them to range between 0.6-1.1 suggesting slight under/overdispersion.

Using an AIC model selection approach as previously implemented, we compared the best-fitting models for every offspring trait with a model including an interaction between maternal age and paternal age to determine whether parental age effects on offspring traits were dependent on the age of the partner. Due to model complexity in investigating these effects on offspring LBS and LRS traits (necessitating the inclusion of a 3-way interaction between (smooth) maternal age, (linear) paternal age and (categorical) offspring sex terms), we investigated the interaction between maternal and paternal age in male and female offspring reproductive traits separately using a *ti* (tensor product interaction) smooth between maternal and paternal age terms. Due to convergence issues, we could not include a random effect of paternal ID in the male and female offspring LBS, LRS models. Full model estimates testing maternal age x paternal age interactions are reported in Table S11.

Since effects on offspring LBS or LRS might be driven by both survival and reproduction, we re-ran the best-fitting offspring lifetime reproduction models to include offspring longevity (lifespan) as an additional fixed covariate. This allowed us to test for effects of parental age on offspring LBS and LRS, over and above any effects of longevity. The other fixed and random effects in these models remained the same.

To determine whether observed maternal age effects on male offspring LBS was being driven by effects on the reproductive ageing pattern in offspring, we re-ran the male offspring annual breeding success models including an interaction between maternal age (continuous) and offspring age (continuous) specified using a *ti* (tensor product interaction) smooth. The other fixed and random effects were the same as in the previous annual models. We compared AIC scores to determine whether including this interaction term improved model fit compared to the base model. We also similarly tested for any maternal age effects on reproductive ageing of daughters by including an interaction between maternal age and offspring age (as specified above) in both the female offspring annual breeding probability and twinning probability models. We could not test for paternal age effects on reproductive ageing in male and female offspring annual reproductive traits due to model convergence issues when also including an interaction between paternal age and offspring age in the aforementioned models.

Ongoing sampling of individuals in long-term studies could potentially introduce bias in our dataset whereby offspring born in more recent years may appear shorter-lived than those born earlier on in the study. To account for any temporal trends in offspring lifespan, we re-ran the offspring lifespan model including birth year as a continuous fixed effect. We found no effect of birth year (estimate: 0.007; 95% CI: -0.007 – 0.021) and found no qualitative differences in the main results (not shown).

For all models, estimation and predictions were carried out and visualized using functions in the R packages, ‘mgcv’, ‘gratia’, ‘ggplot2’, ‘emmeans’ (Wood et al., 2012; Simpson & Singmann., 2018; Wickham, 2016; Lenth., 2018 ). For the models of lifetime traits, the slopes were predicted with fixed effects set as follows: litter size at birth=singleton, parental age class=adults, and average longevity of mothers (8.901 years) and fathers (5.545 years). All analyses were performed in R v.4.4.0 using RStudio v.2024.4.1.748 (R Core Team., 2024; Posit Team., 2024). N.B. In all supplementary tables of models, the terms ‘mat’ and ‘pat’ are short for maternal and paternal respectively.

References

Wood, S. (2012). mgcv: Mixed GAM Computation Vehicle with GCV/AIC/REML smoothness estimation

Simpson, G. L., & Singmann, H. (2018). Package gratia. Ggplot-based graphics and other useful functions for GAMs fitted using Mgcv, 0.1-0 (Ggplot-based graphics and utility functions for working with GAMs fitted using the mgcv package).

Wickham, H., Chang, W., & Wickham, M. H. (2016). Package ‘ggplot2’. Create elegant data visualisations using the grammar of graphics. Version, 2(1), 1-189.

Lenth, R., & Lenth, M. R. (2018). Package ‘lsmeans’. The American Statistician, 34(4), 216-221

R Core Team (2024). _R: A Language and Environment for Statistical Computing_. R Foundation for Statistical Computing, Vienna, Austria. <https://www.R-project.org/>.

Posit team (2024). RStudio: Integrated Development Environment for R. Posit Software, PBC, Boston, MA. URL http://www.posit.co/.

**Supplementary Figures**

Figure S1: Boxplots showing the association between A) maternal and paternal age; B) offspring lifespan and offspring LBS and C) offspring lifespan and offspring LRS (n=1286 offspring individuals). Points represent raw data.


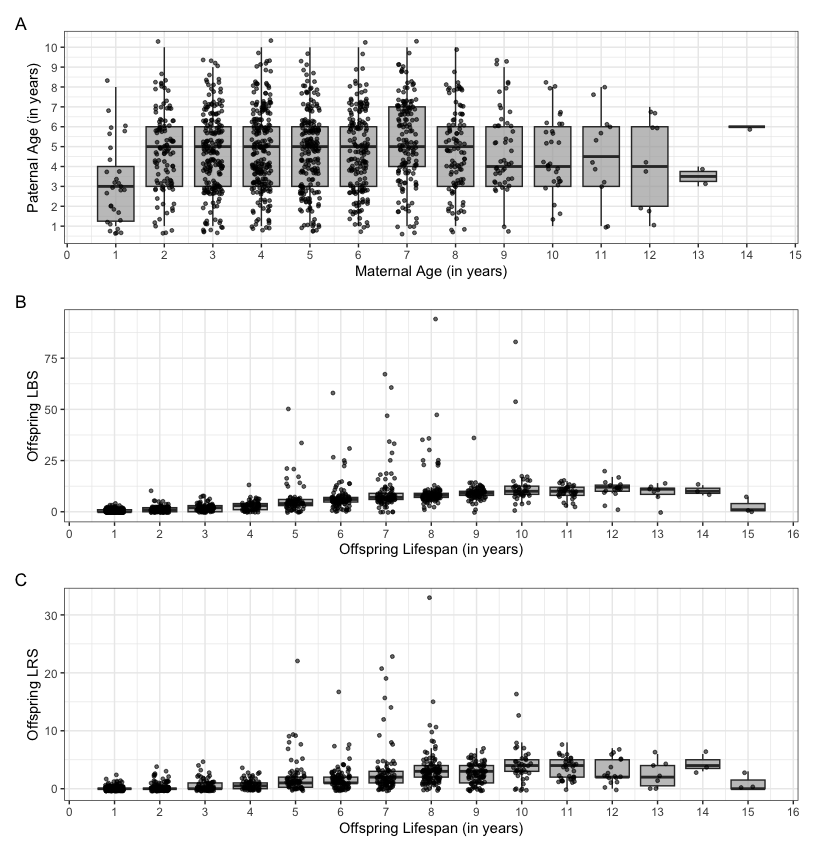


Figure S2: Histogram depicting distributions of all offspring traits. A, B, C, D are categorized by offspring sex. The dotted lines represent the sex-specific average for each offspring trait. E, F, G are distributions for annual breeding performance of male and female offspring shown separately.


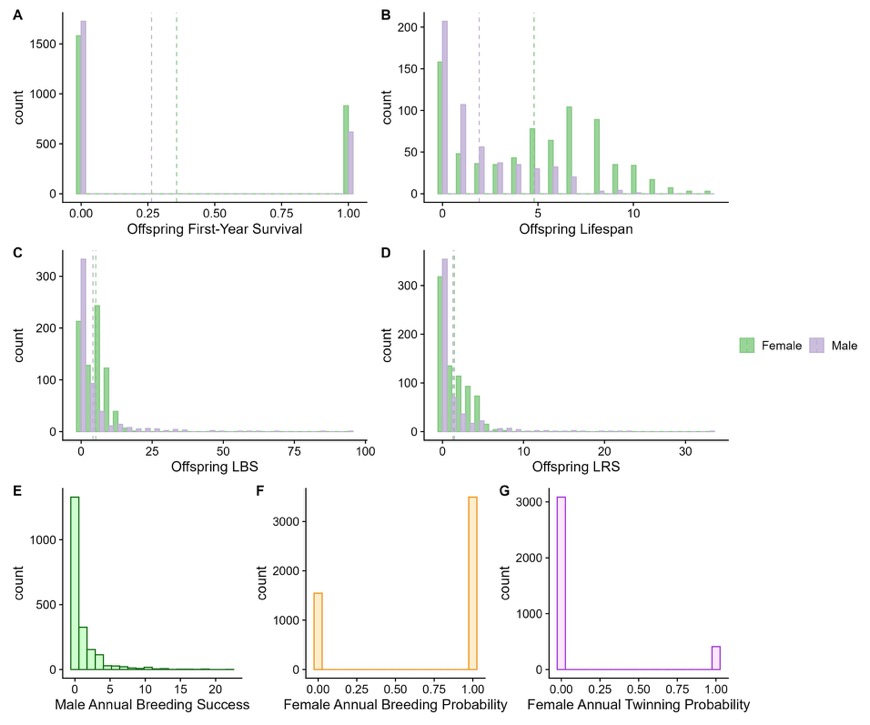


Figure S3: Maternal age effects on offspring first-year survival probability (Table S4). A) represents maternal age effects with the raw data means and standard errors (by sex) alongside model predictions and 95% CIs. B) are the simultaneous 95% CIs for the smooth maternal age terms in the offspring first-year survival model. Highlighted (red and blue) areas indicate maternal ages where offspring first-year survival significantly increases or declines with maternal age. Observations with maternal ages >11 were binned into age 11.


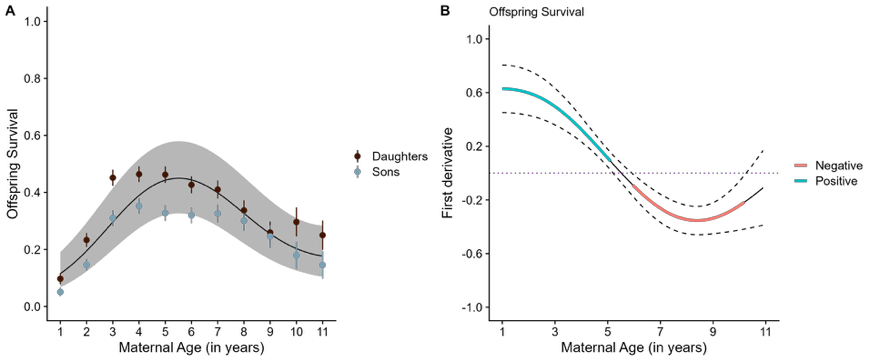


Figure S4: Maternal and paternal age effects on offspring LRS. A) and B) represent maternal and paternal age effects in sons and daughters (Table S5 – Model A). Model predictions, 95% CIs and raw data shown. Jittered raw data only shown for offspring LRS ranging from 0 to 8. Observations with maternal ages >11 were binned into age 11, and paternal ages >9 were binned into age 9. C) and D) represent the simultaneous 95% CIs for the smooth maternal age terms. Highlighted (red and blue) areas indicate maternal ages where offspring LRS significantly increases or declines with maternal age.


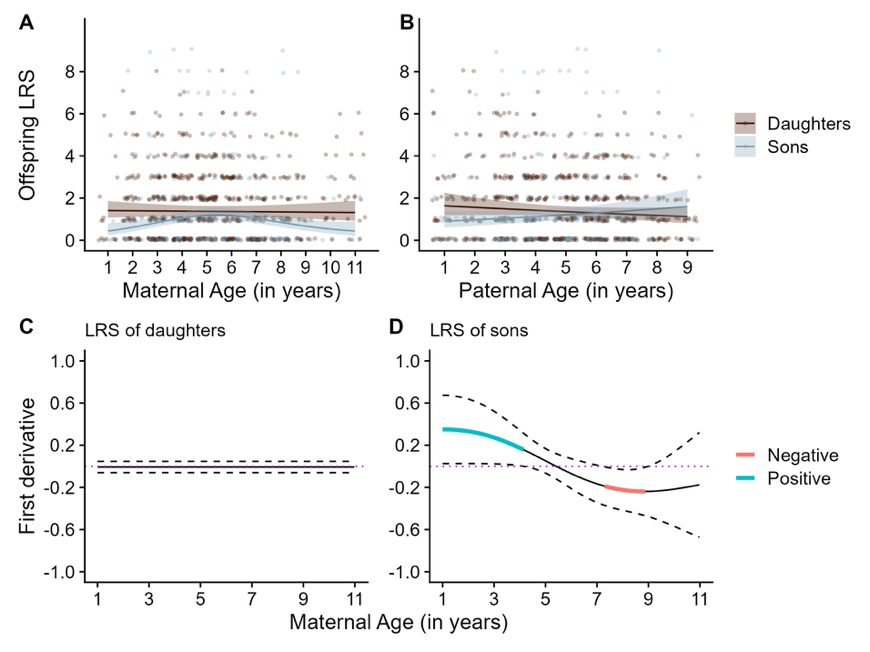


Figure S5: Maternal age effect on annual breeding success of male offspring (n=2064 observations of 532 individuals). Model predictions and 95% CIs at different maternal ages (ages 1,5,9) are displayed alongside raw data (Table S8-Model A). Jittered raw data only shown for offspring annual breeding success ranging from 0 to 8. Observations with offspring ages >9 were binned into age 9.


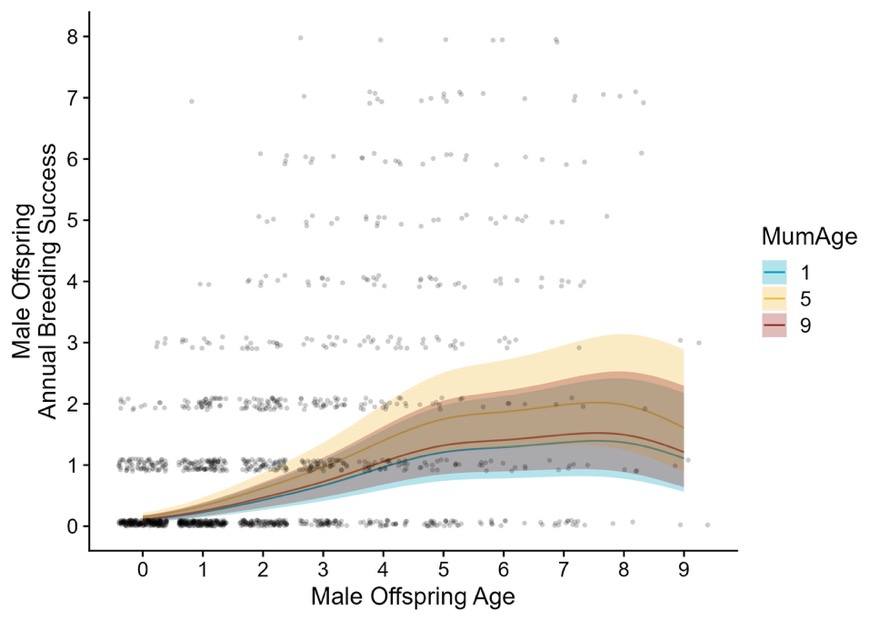


**Supplementary Tables**

Table S1: Sample sizes for each offspring trait: the number of observations or offspring individuals, mothers and fathers.

| Offspring Trait | #Observations | #Individuals | #Mothers | #Fathers |
| --- | --- | --- | --- | --- |
| Offspring First – Year Survival | 4807 | 4807 | 1119 | 851 |
| Offspring Lifetime Breeding Success | 1286 | 1286 | 607 | 429 |
| Offspring Lifetime Recruitment Success | 1286 | 1286 | 607 | 429 |
| Offspring Adult Lifespan | 1286 | 1286 | 607 | 429 |
| Male Offspring Annual Breeding Success | 2064 | 532 | 356 | 267 |
| Female Offspring Annual Breeding Prob | 5041 | 754 | 477 | 323 |
| Female Offspring Annual Twinning Prob | 3495 | 647 | 421 | 292 |

Table S2: Offspring individuals grouped by sex, litter size at birth and parental characteristics across the different offspring traits.

|  | Offspring Sex: | Litter Size at Birth: | Conceived by: | Conceived by: |
| --- | --- | --- | --- | --- |
| Trait: Offspring First – Year Survival  4807 offspring individuals | Males: 48.76%  Females: 51.24% | Twins: 21.18%  Singletons: 78.82% | Lamb ewe: 9.42%  Adult ewe: 90.58% | Lamb ram: 6.28%  Adult ram: 93.72% |
|  |  |  |  |  |
| Traits: Offspring Lifespan, LBS, LRS, Annual Reproduction  1286 offspring individuals | Males: 41.37%  Females: 58.63% | Twins: 13.69%  Singletons: 86.31% | Lamb ewe: 2.33%  Adult ewe: 97.67% | Lamb ram: 5.91%  Adult ram: 94.09% |

Table S3: AIC model selection for offspring sex-specific maternal and paternal age effects across different offspring life-history traits. The best-fitting model is highlighted in bold.

|  | ∆AIC | ∆Residual Deviance | ∆DF |
| --- | --- | --- | --- |
| Offspring First-Year Survival |  |  |  |
| **Base Model** |  |  |  |
| a) + Maternal Age Smooth: Offspring Sex | +4.826 | +2.0 | +0.576 |
| b) + Paternal Age: Offspring Sex | +0.852 | -1.5 | +1.268 |
| c) + Maternal Age Smooth: Offspring Sex + Paternal Age: Offspring Sex | +5.590 | +0.4 | +1.855 |
|  |  |  |  |
| Offspring Adult Lifespan |  |  |  |
| Base Model |  |  |  |
| a) **+ Maternal Age Smooth: Offspring Sex** | -7.617 | -8.3 | +0.300 |
| b) + Paternal Age: Offspring Sex | +1.126 | -0.9 | +1.023 |
| c) + Maternal Age Smooth: Offspring Sex + Paternal Age: Offspring Sex | -6.450 | -9.2 | +1.340 |
|  |  |  |  |
| Offspring LBS |  |  |  |
| Base Model |  |  |  |
| a) + Maternal Age Smooth: Offspring Sex | -8.126 | +6.9 | -11.751 |
| b) + Paternal Age: Offspring Sex | -3.331 | -8.2 | +3.380 |
| c) **+ Maternal Age Smooth: Offspring Sex + Paternal Age: Offspring Sex** | -11.238 | -1.4 | -8.167 |
|  |  |  |  |
| Offspring LRS |  |  |  |
| Base Model |  |  |  |
| a) + Maternal Age Smooth: Offspring Sex | -7.271 | -2.9 | -5.641 |
| b) + Paternal Age: Offspring Sex | -5.420 | -0.9 | -4.212 |
| c) **+ Maternal Age Smooth: Offspring Sex + Paternal Age: Offspring Sex** | -11.299 | -3.2 | -9.334 |
|  |  |  |  |

Table S4: Results from the best-fitting generalized additive mixed model (GAMM) estimating parental age effects on offspring first-year survival. This was fitted using binomial error distribution. Fixed effects, smoothing splines and random effect estimates, 95% confidence intervals and p-values are shown. Results presented are on logit link scale (n=4807 offspring individuals). Statistically significant effects (p < 0.05) are highlighted in bold.

|  | Model | | |
| --- | --- | --- | --- |
| Fixed Effect | Estimate | 95% CI | p-value |
| (Intercept) | -0.678 | -1.374, 0.017 | 0.056 |
| Pat Age | -0.037 | -0.093, 0.019 | 0.194 |
| **Offspring sex (male)** | **-0.592** | **-0.747, -0.437** | **<0.001** |
| **Twin (1)** | **-1.383** | **-1.607, -1.160** | **<0.001** |
| **Mat Lifespan** | **0.047** | **0.007, 0.087** | **0.020** |
| Pat Lifespan | 0.029 | -0.021, 0.080 | 0.249 |
| **Mat Age Class (Yearling mum)** | **-1.064** | **-1.565, -0.563** | **<0.001** |
| **Pat Age Class (Yearling dad)** | **-0.419** | **-0.814, -0.025** | **0.037** |
| Smooth Terms | EDF | χ2 | p-value |
| **s(Mat Age)** | **2.923** | **110.35** | **<0.001** |
| **s(Mat ID)** | **183.581** | **255.11** | **<0.001** |
| **s(Pat ID)** | **53.105** | **82.34** | **0.030** |
| **s(Birth Year)** | **31.277** | **864.46** | **<0.001** |
|  | | | |

Table S5: Results from the generalized additive mixed model (GAMM) estimating parental age effects on offspring lifetime recruitment success. This was fitted using negative binomial error distribution. Fixed effects, smoothing splines and random effect estimates, 95% confidence intervals and p-values are shown. Model A is the best-fitting model and model B includes offspring lifespan as an additional fixed effect. Results presented are on log link scale (n=1286 offspring individuals). Statistically significant effects (p < 0.05) are highlighted in bold.

|  | Model A | | | | Model B | | |
| --- | --- | --- | --- | --- | --- | --- | --- |
| Fixed Effect | Estimate | 95% CI | | p-value | Estimate | 95% CI | p-value |
| (Intercept) | -0.197 | -0.785, 0.391 | | 0.511 | **-2.195** | **-2.694, -1.696** | **<0.001** |
| Pat Age | -0.047 | -0.116, 0.023 | | 0.187 | 0.031 | -0.025, 0.086 | 0.281 |
| **Sex (Male)** | **-0.966** | **-1.452, -0.480** | | **<0.001** | **0.739** | **0.316, 1.162** | **0.001** |
| **Twin (1)** | **-0.425** | **-0.711, -0.140** | | **0.003** | -0.232 | -0.471, 0.006 | 0.056 |
| **Mat Lifespan** | **0.062** | **0.018, 0.105** | | **0.006** | 0.020 | -0.016, 0.055 | 0.276 |
| Pat Lifespan | 0.022 | -0.034, 0.078 | | 0.436 | -0.030 | -0.073, 0.013 | 0.175 |
| Mat Age Class (Yearling mum) | -0.568 | -1.306, 0.170 | | 0.131 | -0.104 | -0.719, 0.511 | 0.740 |
| Pat Age Class (Yearling dad) | -0.147 | -0.598, 0.303 | | 0.521 | -0.087 | -0.453, 0.278 | 0.639 |
| **Pat Age : Offspring Sex (Male)** | **0.118** | **0.026, 0.210** | | **0.012** | 0.035 | -0.042, 0.112 | 0.375 |
| **Offspring Lifespan** |  |  | |  | **0.387** | **0.359, 0.414** | **<0.001** |
| Smooth Terms | EDF | χ2 | | p-value | EDF | χ2 | p-value |
| s(Mat Age) | 1.003 | 0.068 | | 0.801 | 1.000 | 0.937 | 0.333 |
| **s(Mat Age):Offspring Sex (Male)** | **2.489** | **12.873** | | **0.011** | 1.931 | 4.428 | 0.164 |
| **s(Mat ID)** | **85.493** | **103.776** | | **0.003** | **54.785** | **64.916** | **0.009** |
| s(Pat ID) | 30.137 | 34.756 | | 0.150 | 0.742 | 0.737 | 0.501 |
| **s(BirthYear)** | **14.585** | **43.172** | | **<0.001** | **10.004** | **17.165** | **0.032** |
|  |  | |  |  |  |  |  |

Table S6: Slopes for paternal age effects in sons and daughters for offspring lifetime reproductive traits

Estimate (95% CI)

| Slope for paternal age in | Offspring LBS | Offspring LRS |
| --- | --- | --- |
| Sons | 0.044 (-0.024 – 0.112) | 0.071 (-0.014 – 0.157) |
| Daughters | -0.031 (-0.086 – 0.025) | -0.047 (-0.116 –0.023) |

Table S7: AIC model selection to determine inclusion of maternal and paternal age interaction term compared to best-fitting model across different offspring life-history traits. The best-fitting model is highlighted in bold.

|  | ∆AIC | ∆Residual Deviance | ∆DF |
| --- | --- | --- | --- |
| 1. Offspring First-Year Survival |  |  |  |
| **Best-fitting Model (from Table S3)** |  |  |  |
| + Maternal Age Smooth: Paternal Age | +0.669 | -3.0 | +2.756 |
|  |  |  |  |
| 1. Offspring Adult Lifespan |  |  |  |
| **Best-fitting Model (from Table S3)** |  |  |  |
| + Maternal Age Smooth: Paternal Age | +1.011 | -0.8 | +0.918 |
|  |  |  |  |
| 1. Male Offspring LBS |  |  |  |
| **Base Model** |  |  |  |
| + Maternal Age Smooth: Paternal Age | +3.729 | -7.7 | +5.741 |
|  |  |  |  |
| 1. Female Offspring LBS |  |  |  |
| **Base Model** |  |  |  |
| + Maternal Age Smooth: Paternal Age | +2.020 | +0.1 | +0.912 |
|  |  |  |  |
| 1. Male Offspring LRS |  |  |  |
| **Base Model** |  |  |  |
| + Maternal Age Smooth: Paternal Age | +5.566 | -1.2 | +3.522 |
|  |  |  |  |
| 1. Female Offspring LRS |  |  |  |
| **Base Model** |  |  |  |
| + Maternal Age Smooth: Paternal Age | +1.397 | -0.6 | +0.983 |

Table S8: Results from generalized additive mixed model (GAMM) estimating parental age effects on annual breeding success of sons (n=2064 observations of 532 individuals). This was fitted using negative binomial error distribution. Fixed effects, smoothing splines and random effect estimates, 95% confidence intervals and p-values are shown. Model A shows results of the main model while model B includes an interaction between maternal age and offspring age. Results presented are on log link scale. Statistically significant effects (p < 0.05) are highlighted in bold.

|  | Model A | | | Model B | | |
| --- | --- | --- | --- | --- | --- | --- |
| Fixed Effect | Estimate | 95% CI | p-value | Estimate | 95% CI | p-value |
| (Intercept) | -1.227 | -2.012, -0.441 | **0.002** | -1.227 | -2.013, -0.441 | **0.002** |
| Pat Age | 0.047 | -0.030, 0.123 | 0.230 | 0.046 | -0.030, 0.123 | 0.237 |
| **Twin (1)** | **-0.405** | **-0.746, -0.064** | **0.020** | **-0.398** | **-0.739, -0.056** | **0.022** |
| Mat Lifespan | 0.058 | -0.001, 0.117 | 0.053 | 0.057 | -0.001, 0.116 | 0.055 |
| Pat Lifespan | -0.054 | -0.117, 0.009 | 0.094 | -0.053 | -0.116, 0.011 | 0.103 |
| Mat Age Class (Yearling mum) | -0.934 | -2.592, 0.724 | 0.270 | -0.988 | -2.635, 0.659 | 0.240 |
| Pat Age Class (Yearling dad) | -0.193 | -0.771, 0.384 | 0.512 | -0.199 | -0.776, 0.378 | 0.499 |
| Smooth Terms | EDF | χ2 | p-value | EDF | χ2 | p-value |
| s(Mat Age) | 2.189 | 5.102 | 0.086 |  |  |  |
| **s(Offspring Age)** | **2.834** | **376.274** | **<0.001** |  |  |  |
| ti(Mat Age) |  |  |  | 2.175 | 5.134 | 0.087 |
| **ti(Age)** |  |  |  | **2.828** | **377.199** | **<0.001** |
| ti(Mat Age, Offspring Age) |  |  |  | 1.373 | 2.707 | 0.140 |
| **s(ID)** | **180.9** | **979.897** | **<0.001** | **178.8** | **959.912** | **<0.001** |
| **s(Year)** | **29.56** | **377.686** | **<0.001** | **29.59** | **390.446** | **<0.001** |
| **s(Mat ID)** | 42.34 | 224.333 | 0.053 | **43.62** | **232.359** | **0.044** |
| s(Pat ID) | 0.016 | 0.013 | 0.924 | 0.009 | 0.007 | 0.931 |
| s(BirthYear) | 0.001 | 0.001 | 0.656 | 0.001 | 0.001 | 0.734 |
|  |  |  |  |  |  |  |

Table S9: Results from generalized additive mixed model (GAMM) estimating parental age effects on annual breeding probability of daughters (n=5041 observations of 754 individuals). This was fitted using binomial error distribution. Fixed effects, smoothing splines and random effect estimates, 95% confidence intervals and p-values are shown. Model1 shows results of the main model while model 2 includes an interaction between maternal age and offspring age. Results presented are on logit link scale. Statistically significant effects (p < 0.05) are highlighted in bold.

|  | Model A | | | Model B | | |
| --- | --- | --- | --- | --- | --- | --- |
| Fixed Effect | Estimate | 95% CI | p-value | Estimate | 95% CI | p-value |
| (Intercept) | 0.073 | -0.649, 0.794 | 0.844 | 0.069 | -0.653, 0.792 | 0.851 |
| Pat Age | -0.030 | -0.105, 0.045 | 0.434 | -0.030 | -0.105, 0.045 | 0.434 |
| Twin (1) | -0.166 | -0.523, 0.191 | 0.362 | -0.165 | -0.522, 0.192 | 0.365 |
| Mat Lifespan | 0.049 | -0.005, 0.104 | 0.077 | 0.050 | -0.005, 0.105 | 0.076 |
| **Pat Lifespan** | 0.076 | 0.008, 0.144 | **0.028** | 0.076 | 0.008, 0.144 | **0.028** |
| Mat Age Class (Yearling mum) | -0.350 | -1.213, 0.514 | 0.428 | -0.354 | -1.219, 0.510 | 0.422 |
| Pat Age Class (Yearling dad) | 0.238 | -0.291, 0.768 | 0.377 | 0.238 | -0.291, 0.768 | 0.378 |
| Smooth Terms | EDF | χ2 | p-value | EDF | χ2 | p-value |
| s(Mat Age) | 2.606 | 5.652 | 0.209 |  |  |  |
| **s(Offspring Age)** | **2.986** | **610.420** | **<0.001** |  |  |  |
| ti(Mat Age) |  |  |  | 2.601 | 5.588 | 0.213 |
| **ti(Offspring Age)** |  |  |  | **2.986** | **609.710** | **<0.001** |
| ti(Mat Age, Offspring Age) |  |  |  | 1.056 | 0.099 | 0.874 |
| **s(ID)** | **214.955** | **428.834** | **<0.001** | **215.590** | **430.712** | **<0.001** |
| **s(Year)** | **28.459** | **342.027** | **<0.001** | **28.461** | **342.661** | **<0.001** |
| **s(Mat ID)** | **144.753** | **373.885** | **<0.001** | **144.425** | **373.124** | **<0.001** |
| s(Pat ID) | 16.355 | 23.907 | 0.540 | 16.399 | 24.006 | 0.539 |
| s(BirthYear) | 9.952 | 61.776 | 0.145 | 9.954 | 61.915 | 0.144 |
|  |  |  |  |  |  |  |

Table S10: Results from generalized additive mixed model (GAMM) estimating parental age effects on annual twinning probability of daughters (n=3495 observations of 647 individuals). This was fitted using binomial error distribution. Fixed effects, smoothing splines and random effect estimates, 95% confidence intervals and p-values are shown. Model1 shows results of the main model while model 2 includes an interaction between maternal age and offspring age. Results presented are on logit link scale. Statistically significant effects (p < 0.05) are highlighted in bold.

|  | Model A | | | Model B | | |
| --- | --- | --- | --- | --- | --- | --- |
| Fixed Effect | Estimate | 95% CI | p-value | Estimate | 95% CI | p-value |
| (Intercept) | -2.531 | -3.658, -1.404 | **<0.001** | -2.529 | -3.655, -1.402 | **<0.001** |
| Pat Age | -0.051 | -0.164, 0.062 | 0.375 | -0.052 | -0.165, 0.061 | 0.368 |
| Twin (1) | 0.214 | -0.335, 0.763 | 0.445 | 0.228 | -0.323, 0.779 | 0.417 |
| Mat Lifespan | -0.017 | -0.106, 0.071 | 0.699 | -0.017 | -0.106, 0.072 | 0.709 |
| Pat Lifespan | 0.030 | -0.078, 0.139 | 0.583 | 0.030 | -0.079, 0.138 | 0.592 |
| Mat Age Class (Yearling mum) | 0.036 | -1.400, 1.471 | 0.961 | -0.004 | -1.432, 1.424 | 0.996 |
| Pat Age Class (Yearling dad) | 0.227 | -0.586, 1.040 | 0.584 | 0.217 | -0.596, 1.030 | 0.601 |
| Smooth Terms | EDF | χ2 | p-value | EDF | χ2 | p-value |
| s(Mat Age) | 1.000 | 3.131 | 0.077 |  |  |  |
| **s(Offspring Age)** | **2.784** | **145.902** | **<0.001** |  |  |  |
| ti(Mat Age) |  |  |  | 1.000 | 1.185 | 0.276 |
| **ti(Offspring Age)** |  |  |  | **2.789** | **141.912** | **<0.001** |
| ti(Mat Age,Offspring Age) |  |  |  | 1.000 | 2.655 | 0.103 |
| s(ID) | 81.83 | 136.587 | 0.166 | 84.90 | 143.630 | 0.133 |
| **s(Year)** | **14.44** | **31.597** | **0.006** | **14.78** | **33.324** | **0.004** |
| **s(Mat ID)** | **107.6** | **248.751** | **0.003** | **106.4** | **245.166** | **0.003** |
| s(Pat ID) | 24.93 | 43.067 | 0.473 | 23.67 | 39.901 | 0.497 |
| s(BirthYear) | 0.001 | 0.000 | 0.999 | 0.002 | 0.000 | 0.999 |
|  |  |  |  |  |  |  |

Table S11: Results from generalized additive mixed model (GAMM) estimating an interaction between maternal age and paternal age on offspring traits. Fixed effects, smoothing splines and random effect estimates, 95% confidence intervals and p-values are shown. Results presented are on link scale. Statistically significant effects (p < 0.05) are highlighted in bold.

1. Offspring First-Year Survival (Binomial)

| Fixed Effect | Estimate | 95% CI | p-value |
| --- | --- | --- | --- |
| (Intercept) | -0.649 | -1.346, 0.047 | 0.068 |
| Pat Age | -0.038 | -0.094, 0.018 | 0.180 |
| **Offspring Sex (Male)** | **-0.594** | **-0.749, -0.439** | **<0.001** |
| **Twin (1)** | **-1.388** | **-1.612, -1.164** | **<0.001** |
| **Mat Lifespan** | **0.046** | **0.007, 0.086** | **0.022** |
| Pat Lifespan | 0.027 | -0.023, 0.077 | 0.289 |
| **Mat Age Class (Yearling mum)** | **-1.113** | **-1.621, -0.605** | **<0.001** |
| **Pat Age Class (Yearling dad)** | **-0.430** | **-0.824, -0.036** | **0.032** |
| Smooth Terms | EDF | χ2 | p-value |
| **s(Mat Age)** | **2.923** | **111.088** | **<0.001** |
| ti(Mat Age, Pat Age) | 1.738 | 1.969 | 0.389 |
| **s(Mat ID)** | **183.972** | **255.179** | **<0.001** |
| **s(Pat ID)** | **53.251** | **82.086** | **0.029** |
| **s(Birth Year)** | **31.281** | **863.263** | **<0.001** |
|  | | | |

1. Offspring Lifespan (Negative Binomial)

| Fixed Effect | Estimate | 95% CI | p-value |
| --- | --- | --- | --- |
| **(Intercept)** | **1.070** | **0.719, 1.420** | **<0.001** |
| Pat Age | -0.037 | -0.076, 0.002 | 0.062 |
| **Offspring Sex (Male)** | **-0.947** | **-1.067, -0.827** | **<0.001** |
| Twin (1) | -0.151 | -0.324, 0.021 | 0.086 |
| **Mat Lifespan** | **0.039** | **0.013, 0.066** | **0.003** |
| **Pat Lifespan** | **0.043** | **0.008, 0.077** | **0.015** |
| Mat Age Class (Yearling mum) | -0.325 | -0.770, 0.121 | 0.153 |
| Pat Age Class (Yearling dad) | -0.019 | -0.298, 0.259 | 0.891 |
| Smooth Terms | EDF | χ2 | p-value |
| s(Mat Age): Offspring Sex (Female) | 1.008 | 0.505 | 0.478 |
| **s(Mat Age):Offspring Sex (Male)** | **2.429** | **14.018** | **0.006** |
| ti(Mat Age, Pat Age) | 1.000 | 1.091 | 0.296 |
| s(Mat ID) | 0.003 | 0.003 | 0.994 |
| s(Pat ID) | 0.003 | 0.003 | 0.961 |
| **s(Birth Year)** | **17.982** | **69.679** | **<0.001** |
|  | | | |

1. Male Offspring LBS (Negative Binomial)

| Fixed Effect | Estimate | 95% CI | p-value |
| --- | --- | --- | --- |
| (Intercept) | -0.588 | -1.652, 0.477 | 0.279 |
| Pat Age | 0.033 | -0.081, 0.147 | 0.569 |
| **Twin (1)** | **-0.536** | **-1.023, -0.050** | **0.031** |
| **Mat Lifespan** | **0.098** | **0.014, 0.182** | **0.022** |
| Pat Lifespan | 0.042 | -0.053, 0.137 | 0.384 |
| Mat Age Class (Yearling mum) | -1.134 | -3.073, 0.804 | 0.251 |
| Pat Age Class (Yearling dad) | -0.079 | -0.916, 0.758 | 0.853 |
| Smooth Terms | EDF | χ2 | p-value |
| **s(Mat Age)** | **2.52** | **19.610** | **<0.001** |
| ti(Mat Age, Pat Age) | 4.12 | 7.156 | 0.157 |
| **s(Mat ID)** | **75.70** | **95.566** | **0.003** |
| **s(Birth Year)** | **12.24** | **33.519** | **0.001** |
|  | | | |

1. Female Offspring LBS (Negative Binomial)

| Fixed Effect | Estimate | 95% CI | p-value |
| --- | --- | --- | --- |
| **(Intercept)** | **1.131** | **0.753, 1.508** | **<0.001** |
| Pat Age | -0.031 | -0.074, 0.012 | 0.156 |
| Twin (1) | -0.117 | -0.318, 0.085 | 0.257 |
| **Mat Lifespan** | **0.039** | **0.009, 0.068** | **0.010** |
| **Pat Lifespan** | **0.041** | **0.002, 0.080** | **0.041** |
| Mat Age Class (Yearling mum) | -0.235 | -0.765, 0.296 | 0.385 |
| Pat Age Class (Yearling dad) | 0.089 | -0.222, 0.400 | 0.575 |
| Smooth Terms | EDF | χ2 | p-value |
| s(Mat Age) | 2.432 | 6.375 | 0.109 |
| ti(Mat Age, Pat Age) | 1.358 | 0.550 | 0.767 |
| s(Mat ID) | 0.005 | 0.000 | 0.975 |
| **s(Birth Year)** | **9.128** | **15.385** | **0.020** |
|  | | | |

1. Male Offspring LRS (Negative Binomial)

| Fixed Effect | Estimate | 95% CI | p-value |
| --- | --- | --- | --- |
| **(Intercept)** | **-1.511** | **-2.779, -0.242** | **0.020** |
| Pat Age | 0.064 | -0.075, 0.202 | 0.368 |
| **Twin (1)** | **-0.673** | **-1.273, -0.073** | **0.028** |
| **Mat Lifespan** | **0.129** | **0.029, 0.230** | **0.011** |
| Pat Lifespan | -0.002 | -0.118, 0.114 | 0.973 |
| Mat Age Class (Yearling mum) | -1.211 | -3.776, 1.355 | 0.355 |
| Pat Age Class (Yearling dad) | -0.436 | -1.493, 0.621 | 0.419 |
| Smooth Terms | EDF | χ2 | p-value |
| s(Mat Age) | 2.171 | 5.919 | 0.089 |
| ti(Mat Age, Pat Age) | 1.544 | 1.722 | 0.483 |
| s(Mat ID) | 25.724 | 25.545 | 0.522 |
| **s(Birth Year)** | **5.556** | **7.249** | **0.185** |
|  | | | |

1. Female Offspring LRS (Negative Binomial)

| Fixed Effect | Estimate | 95% CI | p-value |
| --- | --- | --- | --- |
| (Intercept) | 0.097 | -0.426, 0.620 | 0.716 |
| Pat Age | -0.059 | -0.118, 0.000 | 0.052 |
| Twin (1) | -0.243 | -0.528, 0.041 | 0.093 |
| Mat Lifespan | 0.032 | -0.008, 0.073 | 0.113 |
| Pat Lifespan | 0.037 | -0.016, 0.091 | 0.171 |
| Mat Age Class (Yearling mum) | -0.255 | -0.885, 0.375 | 0.427 |
| Pat Age Class (Yearling dad) | -0.091 | -0.516, 0.333 | 0.673 |
| Smooth Terms | EDF | χ2 | p-value |
| s(Mat Age) | 1.000 | 0.056 | 0.814 |
| ti(Mat Age, Pat Age) | 1.001 | 0.586 | 0.445 |
| s(Mat ID) | 0.014 | 0.012 | 0.915 |
| **s(Birth Year)** | **16.294** | **45.455** | **<0.001** |
|  | | | |
